# Meta-Analysis of *RAGE* Gene Polymorphism and Coronary Heart Disease Risk

**DOI:** 10.1371/journal.pone.0050790

**Published:** 2012-12-06

**Authors:** Jun Wang, Lianjiang Zou, Zhigang Song, Xilong Lang, Shengdong Huang, Fanglin Lu, Lin Han, Zhiyun Xu

**Affiliations:** Department of Cardiothoracic Surgery, Changhai Hospital, Second Medical University, Shanghai, People’s Republic of China; University of Milan, Italy

## Abstract

**Background:**

Recent data from human and animal studies have shown an upregulated expression of advanced glycosylation end product–specific receptor (RAGE) in human atherosclerotic plaques 1 and in retina, messangial, and aortic vessels, suggesting an important role of RAGE in the pathogenesis of atherothrombotic diseases. In the past few years, the relationship between *RAGE* polymorphisms (−429T/C, −374T/A, and G82S) and coronary heart disease (CHD) has been reported in various ethnic groups; however, these studies have yielded contradictory results.

**Methods:**

PubMed, ISI web of science, EMBASE and the Chinese National Knowledge Infrastructure databases were systematically searched to identify relevant studies. Data were abstracted independently by two reviewers. A meta-analysis was performed to examine the association between *RAGE* polymorphisms and susceptibility to CHD. Odds ratios (ORs) and 95% confidence intervals (95% CIs) were calculated.

**Results:**

A total of 17 studies including 4343 patients and 5402 controls were involved in this meta-analysis. Overall, no significant results were observed for −429T/C (OR  = 1.01, 95% CI: 0.92–1.12, P  = 0.78), −374T/A (OR  = 1.11, 95% CI: 0.98–1.26, P  = 0.09) and G82S (OR  = 1.12, 95% CI: 0.86–1.45, P  = 0.41) polymorphism. In the stratified analyses according to ethnicity, sample size, CHD endpoint and Hardy-Weinberg status, no evidence of any gene-disease association was obtained.

**Conclusions:**

This meta-analysis demonstrates that there is no association between the RAGE −429T/C, −374T/A and G82S polymorphisms and CHD.

## Introduction

Coronary heart disease (CHD), including its most severe complication, myocardial infarction, is the leading cause of death in the industrialized world. Traditional risk such as hypertension, diabetes mellitus, dyslipidemia and smoking can only explain approximately two-thirds of the observed clinical events. This has maintained interest in other biochemical and genetic factors that might contribute to the underlying pathophysiology of vascular disease.

Recently, an important role of receptor for advanced glycation end product (RAGE) in the pathogenesis of vascular diseases has generated great interest. RAGE is a multiligand member of the immunoglobulin superfamily of cell surface molecules [1] and engages diverse ligands relevant to distinct pathologic processes [2]. Irreversible advanced glycation end products (AGEs) are one class of RAGE ligands and occur at an increased level under conditions of hyperglycemia and in inflammatory environments [3], [4]. Sustained interaction with higher levels of AGEs increases receptor expression and activation of proinflammatory and procoagulant pathways [5]–[7], which may be the key factors linking the RAGE system with atherosclerosis [8], [9]. The *RAGE* gene, located on chromosome 6p21.3, contains 11 exons and a 1.7-kb 5′-flanking region [1]. Several variants of the *RAGE* gene, including functional polymorphisms −429T/C (rs1800625) in the promoter region, −374T/A (rs1800624) in intron 7 and G82S (rs2070600) in exon 3, have been implicated in the development of diabetic atherosclerosis [10]–[12].

Despite the biological plausibility of *RAGE* polymorphism as a modulator of CHD susceptibility, previously inconsistent results have appeared in the literature. Published studies have generally been restricted in terms of sample size and ethnic diversity, and individual studies may have insufficient power to achieve a comprehensive and reliable conclusion. We therefore performed a meta-analysis of the published studies to clarify this inconsistency and to establish a comprehensive picture of the relationship between *RAGE* and CHD.

## Materials and Methods

### Literature Search Strategy

The literature included in our analysis was selected from PubMed, ISI web of science, EMBASE and Chinese National Knowledge Infrastructure (CNKI) with keywords relating to the relevant gene (e.g. “receptor for advanced glycation end product”, “RAGE”, “advanced glycosylation end product–specific receptor”, “AGER”) in combination with words related to CHD (e.g. “coronary heart disease”, “coronary artery disease”, “myocardial infarction”, “ischemic heart disease”, “atherosclerosis”, “arteriosclerosis”, and “coronary stenosis”). Genetic association studies published before May 2012 on CHD and polymorphisms in the *RAGE* gene described above were retrieved, and their references were checked to identify other relevant publications. All relevant reports identified were included without language restriction.

### Eligible Studies and Data Extraction

Eligible studies had to meet all of the following criteria: (1) original papers containing independent data published in a peer-reviewed journal; (2) genotype distribution information in cases and controls or odds ratio with its 95% confidence interval and P-value.; (3) case–control or cohort studies; and (4) the diagnosis of CHD patients was confirmed pathologically or angiographically. The major reasons for exclusion of studies were (1) overlapping data and (2) case-only studies, family-based studies and review articles.

Information was carefully extracted from all eligible publications independently by two authors according to the inclusion criteria listed above. The following data were collected from each study: first author, year of publication, diagnosis criterion, ethnicity, Hardy–Weinberg equilibrium (HWE) status, genotyping method, source of control, age, sex, body mass index (BMI), total number of cases and controls and genotype frequency in cases and controls. Relevant clinical outcomes included confirmed MI (generally by WHO criteria) and coronary stenosis (defined as at least 50% stenosis of one or more major coronary arteries on the basis of computer assisted assessments). For studies in which data could not be separated according to type of CHD from published data, cases were classified in the more inclusive category of coronary stenosis for the purpose of subsidiary analyses. Discrepancies in data extraction were resolved by discussion between all authors through consensus. Studies with different ethnic groups were considered as individual studies for our analyses.

### Statistical Methods

Deviation from Hardy–Weinberg equilibrium was examined by Chi-square test. Odds ratio (OR) with 95% confidence intervals (CIs) was used to assess the strength of association between the *RAGE* gene polymorphisms and CHD risk. For −429T/C, −374T/A, and G82S polymorphism, we pooled the OR by comparing risk allele carrier genotypes versus wild-type genotype for low frequency of homozygous variant. Heterogeneity was assessed with standard Q-statistic test. If heterogeneity existed, the random effects model (the DerSimonian and Laird method) [13], which yields wider confidence intervals, was adopted to calculate the overall OR value. Otherwise, the fixed effect model (the Mantel - Haenszel method) was used [14]. The Z test was used to determine the significance of the pooled OR. In addition, sources of heterogeneity were investigated by stratified meta-analyses based on ethnicity, sample size (No. cases <300, or ≥300) and types of CHD end points (myocardial infarction versus coronary stenosis).

In order to assess the stability of the results, one-way sensitivity analyses were performed by removing each individual study in turn from the total and re-analyzing the remainder. Publication bias was assessed using Egger’s test and Begg’s funnel plots [15]. All P values are two-sided at the P  = 0.05 level. Analyses were performed using the Stata software (version 11.0; Stata Corp LP, College Station, TX).

## Results

### Characteristics of Studies

The combined search yielded 101 references. Study selection process was shown in figure S1. A total of 17 studies were finally included with 4343 patients and 5402 controls [16]–[32]. For the −429T/C polymorphism, 13 studies were available, including a total of 3552 cases and 3998 controls. For the −374T/A polymorphism, 8 studies involved a total of 2605 cases and 3008 controls. For the G82S polymorphism, 10 studies involved a total of 2741 cases and 4336 controls. The detailed characteristics of the studies included in this meta-analysis are shown in Table 1 and Table S1.

**Table 1:** Characteristics of the studies included in the meta-analysis.

Study	Year	Ethnicity	Assessment of end point	Endpoint	No. of Case/control	Mean age of case/control	Gender component in case and control (% male)	Control source	Genotyping method
Pettersson-Fernholm [16]	2003	American	≥50% stenosis	CHD	85/872	NA/NA	NA/NA	HB	Minisequencing
Falcone [17]	2004	Italian	≥50% stenosis	CAD	175/350	61.8/59.6	79.4/77.4	HB	RFLP
Kirbis [18]	2004	Slovenian	WHO criteria	MI, CAD	168/241	59.3/66.9	64.9/43.2	HB	RFLP
Santos [19]	2005	Brazilian	WHO criteria	IHD	252/225	61.7/61.3	34.0/50.5	HB	RFLP
Hofmann [20]	2005	American	WHO criteria	MI, CAD	132/1500	661./58.7	67.0/47.0	PB	RFLP
Zee [21]	2006	American	WHO criteria	MI	341/341	61.0/60.8	NA/NA	PB	TaqMan
Yoon [22]	2007	Korean	≥50% stenosis	CAD	747/805	55.7/53.2	NA/NA	HB	TaqMan
Lu [23]	2008	Chinese	≥50% stenosis	CAD	128/357	67.0/61.0	67.2/47.8	PB, HB	Sequencing
Kucukhuseyin [24]	2009	Turkish	≥50% stenosis	CAD	106/53	60.0/58.0	32.5/49.1	PB	RFLP
Peng [25]	2010	Chinese	≥50% stenosis	CAD	370/560	64.0/63.0	63.2/58.1	HB	RFLP
Lu [26]	2010	Chinese	≥50% stenosis	CAD	270/112	63.7/61.8	65.2/58.0	PB	RFLP
Xie [27]	2010	Chinese	≥50% stenosis	CHD	125/88	65.2/62.7	62.2/50.0	HB	RFLP
Gao [28]	2010	Chinese	≥50% stenosis	CAD	330/370	61.8/60.8	72.1/45.3	PB, HB	RFLP
Kucukhuseyin [29]	2011	Turkish	≥50% stenosis	CAD	113/55	60.0/58.0	32.5/49.1	PB	RFLP
Boiocchi [30]	2011	Italian	European Society of Cardiology criteria	MI	691/234	59.0/62.0	83.0/64.0	HB	RFLP
Hou [31]	2011	Chinese	≥50% stenosis	CAD	223/187	57.7/58.2	78.1/77.3	HB	RFLP
Aydogan [32]	2012	Turkish	NA	CAD	87/52	60.0/58.1	NA/NA	PB	RFLP

NA: not available; MI: myocardial infarction; CAD: coronary artery disease; CHD: coronary heart disease; IHD: ischemic heart disease; PB: population based; HB: hospital based; WHO: world health organization.

### Association of −374T/A Polymorphism with CHD

**Figure 1:**
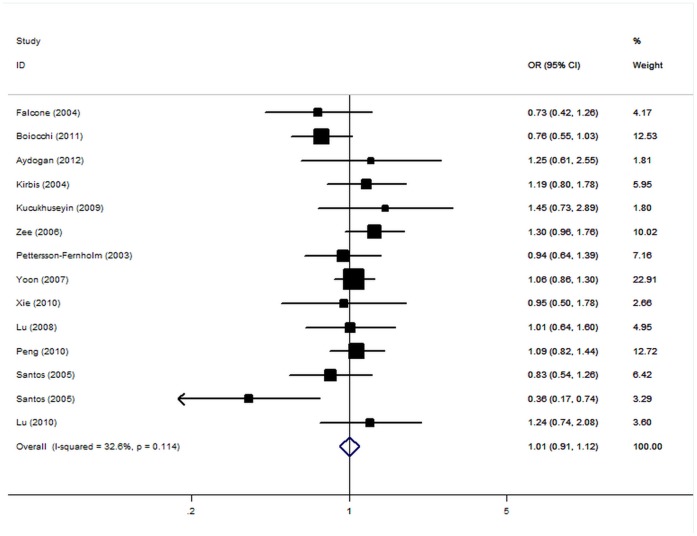
Forest plot from the meta-analysis of coronary heart disease and *RAGE* −374T/A polymorphism.

The crude ORs were performed for A allele carriers versus non-carriers. Individuals carrying the risk A allele were not statistically significant associated with an increased risk to coronary heart disease compared with those non-carriers. The summary OR was 1.01 [95% CI: 0.92–1.12, P(Z)  = 0.78, P(Q) = 0.12; figure 1]. Data from the studies did not exhibit statistically significant heterogeneity in the majority of contrasts. When stratifying for ethnicity, an OR of 0.98 (95% CI: 0.84–1.14) and 1.09 (95% CI: 0.95–1.25) resulted for the risk allele carriers, among Caucasian and Asian, respectively. In the subgroup analyses by CHD end points, the OR for MI of the variant carriers was 0.95 (95% CI: 0.65–1.39) and for coronary stenosis was 1.00 (95% CI: 0.86–1.17). Subsidiary analyses of sample size yielded an OR for small studies of 0.98 (95% CI: 0.83–1.15) and for big studies of 1.04 (95% CI: 0.91–1.19). Subgroup analyses according to HWE status yielded an OR for controls consistent to HWE of 1.01 (95% CI: 0.91–1.13), while no significant results were found for controls deviated from HWE (Table 2).

**Table 2:** Meta-analysis of the *RAGE* polymorphism on coronary heart disease risk.

	−429T/C				−374T/A				G82S			
Sub-group analysis	No. of case/control	OR (95%CI)	P(Z)	P(Q)	No. of case/control	OR (95%CI)	P(Z)	P(Q)	No. of case/control	OR (95%CI)	P(Z)	P(Q)
Total	3552/3998	1.01 (0.92–1.12)	0.78	0.12	2605/3008	1.11 (0.98–1.26)	0.09	0.30	2741/4336	1.12 (0.86–1.45)	0.41	<10^−5^
Ethnicity												
Caucasian	1637/1927	0.98 (0.84–1.14)	0.80	0.12	686/751	1.02 (0.81–1.29)	0.84	0.11	473/1841	0.87 (0.57–1.34)	0.53	0.90
Asian	1840/2015	1.09 (0.95–1.25)	0.24	0.96	1844/2201	1.12 (0.96–1.30)	0.15	0.92	2268/2495	1.17 (0.86–1.58)	0.31	<10^−5^
Sample size												
<300	1391/2069		0.79	0.18	1148/1304	1.22 (1.03–1.45)	0.02	0.50	1283/2633	1.28 (0.92–1.76)	0.14	0.002
≥300	2161/1929	1.04 (0.91–1.19)	0.57	0.10	1457/1704	1.01 (0.85–1.21)	0.91	0.21	1458/1703	0.81 (0.69–0.96)	0.01	0.73
Endpoint												
Stenosis	2467/2491	1.00 (0.86–1.17)	0.06	0.21	2264/2667	1.18 (1.03–1.35)	0.02	0.63	2335/2418	1.17 (0.86–1.58)	0.31	<10^−5^
MI	1085/1507	0.95 (0.65–1.39)	0.79	0.04	341/341	0.80 (0.58–1.11)	0.18	NA	406/1918	0.88 (0.55–1.40)	0.58	0.90
HWE status												
Yes	3424/3641	1.01 (0.91–1.13)	0.78	0.08	2402/2595	1.10 (0.97–1.25)	0.15	0.56	1820/3125	1.18 (0.77–1.80)	0.45	<10^−5^
No	128/357	1.01 (0.64–1.60)	0.96	NA	203/413	1.52 (0.53–4.36)	0.43	0.03	921/1211	1.05 (0.73–1.50)	0.79	0.01

NA: not available.

### Association of −429T/C Polymorphism with CHD

**Figure 2:**
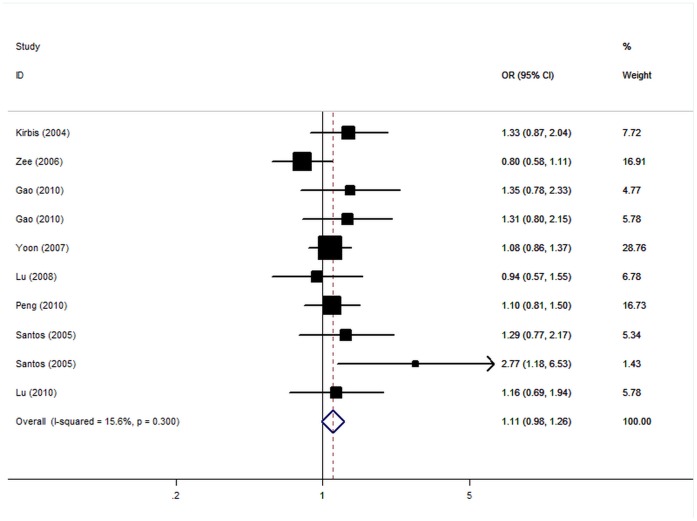
Forest plot from the meta-analysis of coronary heart disease and *RAGE* −429T/C polymorphism.

Overall, the OR of the −429T/C polymorphism for CHD was 1.11 [95% CI: 0.98–1.26; P(Z)  = 0.09, P(Q) = 0.30; figure 2]. In the stratified analysis by ethnicity and HWE status, no significant associations were found (Table 2). After stratification for CHD endpoint, positive results were observed for coronary stenosis with OR of 1.18 (95CI: 1.03–1.35), while no associations were found for MI subgroup. Subsidiary analyses of sample size yielded an OR for small studies of 1.22 (95% CI: 1.03–1.45) and for big studies of 1.01 (95% CI: 0.85–1.21).

### Association of G82S Polymorphism with CHD

**Figure 3:**
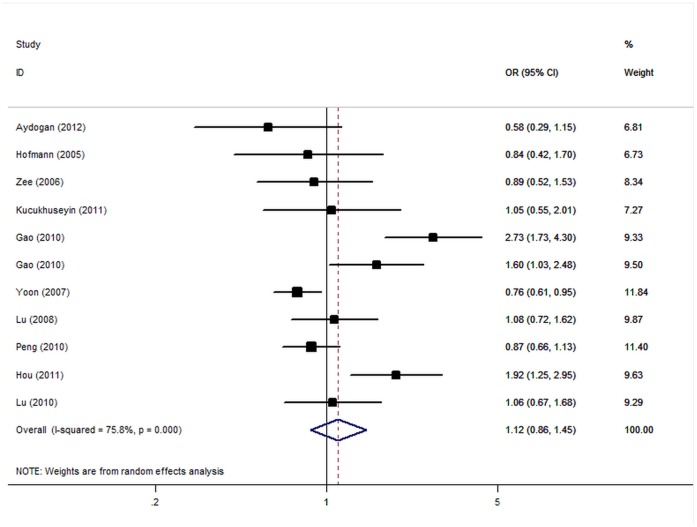
Forest plot from the meta-analysis of coronary heart disease and *RAGE* G82S polymorphism.

In the overall analysis, the S allele was not significantly associated with elevated CHD (OR = 1.12, 95% CI: 0.86–1.45; P(Z)  = 0.41, P(Q) <10^−5^; Figure 3). However, there was significant heterogeneity among included studies (P<0.05). When studies were stratified for ethnicity, CHD endpoint and HWE status among controls, we still did not observe an association between G82S genotype and CHD risk. In the stratified analysis by sample size, no significant associations were also detected in small studies, while only marginal significant associations were detected for big studies (Table 2).

### Haplotype Analyses

Haplotype analyses between −429T/C, −374T/A, and G82S polymorphisms were performed in the 3 studies, involving 1461 cases and 1516 controls. No association between CHD and −429T/C, −374T/A, and G82S polymorphism is supported by the linkage disequilibrium (LD) analyses (Table S2). The pooled ORs of the TTG, TAG, CTG and TTS for CHD were 1.01 (95% CI: 0.92–1.12), 1.10 (95% CI: 0.97–1.25), 0.96 (95% CI: 0.76–1.21) and 0.88 (95% CI: 0.76–1.03), respectively (Table S3).

### Sensitivity Analyses and Publication Bias

A single study involved in the meta-analysis was deleted each time to reflect the influence of the individual dataset to the pooled ORs, and the corresponding pooled ORs were not qualitatively altered, suggesting stability of the meta-analyses. Begg’s funnel plot and Egger’s test were performed to access the publication bias of the literatures. The shape of the funnel plots was symmetrical for these polymorphisms (figure S2, S3 and S4). The statistical results still did not show publication bias in these studies for −374T/A (Egger’s test, P  = 0.37), −429T/C (Egger’s test, P  = 0.07) and G82S (Egger’s test, P  = 0.95).

## Discussion

Large sample and unbiased epidemiological studies of predisposition gene polymorphisms could provide insight into the in vivo relationship between candidate genes and complex diseases. The present meta-analysis provides the first comprehensive assessment of the risk of CHD and three variants on *RAGE* gene. Its strength was based on the accumulation of published data giving greater information to detect significant differences. In total, the meta-analysis involved 17 studies for CHD which provided 4343 cases and 5402 controls.

Through the combined examination of independent studies, we did not find evidence supporting a positive relation between −429T/C, −374T/A, and G82S polymorphism of the *RAGE* gene and CHD susceptibility. Meanwhile, haplotypes analyses of the −429T/C, −374T/A, and G82S polymorphisms reveal the no association between the combination of these alleles and risk of CHD. For the subgroup analysis based on ethnicity, sample size, CHD endpoint and HWE status, we were unable to observe any effect modification, which is in line with the pooled analysis. In addition, data from the studies did not exhibit statistically significant heterogeneity in the majority of contrasts. There are some points should be concerned for the inconsistent results in early reports. Firstly, ethnic differences may attribute to these different results, since the distributions of the RAGE polymorphism were different between various ethnic populations. For instance, the frequencies of *RAGE* −374T/A polymorphism allele differs from 14% in Chinese population [23], [25], 37% in Turkish populations [24], [32], to 44% in Caucasians [17], [29]. On the other hand, study design or small sample size or some environmental factors may affect the results. Most of these studies did not consider most of the important environmental factors. It is possible that variation at this locus has modest effects on CHD, but environmental factors may predominate in the progress of CHD, and mask the effects of this variation. Specific environmental factors like lifestyle and diabetes that have been already well studied in recent decades. It is still unknown whether the lifestyle characteristics of different populations influence the association between genotype and CHD and whether genetic factors influence the ages of onset of CHD. The unconsidered factors mixed together may cover the role of *RAGE* polymorphism. Thus, even if the variation has a causal effect on CHD, it may take a long time to be observed.

If genetic susceptibility to CHD is, in part, mediated through metabolic gene polymorphisms, it is possible that the combinations of certain genotypes may be more discriminating as risk factors for CHD than a single locus genotype. Previously study have found that haplotypes carrying either the −429T/C or the −374T/A variant are associated with reduced risk of MI and ischemic stroke, respectively, suggesting a protective role for *RAGE* promoter gene polymorphisms in atherothrombosis [21]. In addition, Gao et al suggested that a haplotype bearing the G82S variant was significantly associated with an increased risk of CAD [28]. Unfortunately, most of studies included in present meta-analysis did not explore the interaction between serum RAGE haplotype and CHD. Our results also suggest the importance of including a haplotype-based approach for assessment of genetic association investigations. Haplotype-based case–control studies are warranted to confirm our findings in the future.

Advanced glycation end products (AGEs) show their effects in extracellular matrix, in vascularate and by binding to their receptors (RAGE). Because of their prothrombotic and proinflammatory effects of RAGEs, the single nucleotide polymorphisms of *RAGE* gene is getting important for being the candidate gene for atherosclerosis and the other associated diseases. In addition, −429T/C and −374T/A gene variants have been shown to have a 2-fold and 3-fold increase in transcriptional activity, respectively [33]. As for Gly82Ser variant, its location in V-domain of the extracellular segment of the receptor in exon 3 has been demonstrated to exhibit differential receptor-binding affinity for AGER ligands [34]. However, the pathophysiological consequences of the altered transcriptional activity or receptor-binding affinity associated with these gene variants remain elusive.

Endogenous secretory RAGE (esRAGE) is an endogenous splicing RAGE variant. Administration of soluble RAGE isoforms inhibited atherogenesis and mitigated further progression of atherosclerotic lesions in diabetic mice [35], [36]. Clinical studies have shown that serum esRAGE level was decreased in patients with diabetes and cardiovascular disease, and reduced circulating esRAGE level predicted cardiovascular mortality in patients with end-stage renal disease [37]. Yet, comparatively little was known about the mechanism of production and secretion of esRAGE. Previously studies implied that mechanisms evolving in regulating RAGE expression may take part in the regulation of esRAGE expression in vivo [25], [38]. Further studies are needed to explore the mechanism of how RAGE polymorphism regulated esRAGE expression.

In interpreting the result s, some limitations of this meta-analysis should be addressed. Firstly, lacking of individual-level data prevent us from making further analysis to identify any interactions between genetic variation and metabolic traits. Secondly, in the subgroup analyses, different ethnicities were pooled in the Asian ethnic group which may bring in some heterogeneity. As studies among specific ethnic group are currently limited, further studies including a wider spectrum of subjects should be carried to investigate the role of these variants in different populations. Thirdly, the overall outcomes were based on individual unadjusted ORs, while a more precise evaluation should be adjusted by other potentially suspected factors including age, sex, diabetes status, and environmental factors. Finally, because of the complex nature of CHD, it is unlikely that a SNP in one single gene would be obviously associated with an increase in asthma risk, without consideration of any other polymorphic susceptible genes.

To conclude, our meta-analysis did not support an association of the −429T/C, −374T/A, and G82S polymorphism of *RAGE* with CHD. The importance of these polymorphisms as a predictor of the risk of CHD is probably very small and the screening utility of this genetic variant in asymptomatic individuals may not be warranted. It is also known that the pathogenesis of CHD is complex and polygenetic in the vast majority of patients, with several genes, each with a small to moderate effect, acting individually, together or in association with important environmental determinants. Larger studies of different ethnic populations, especially with detailed individual information, are needed to confirm our findings.

## Supporting Information

Figure S1 **The flow chart of the included studies.**
(TIFF)

Figure S2 **Begg’s funnel plot of** ***RAGE*** **−374T/A polymorphism and coronary heart disease risk.**
(TIFF)

Figure S3 **Begg’s funnel plot of** ***RAGE*** **−429T/C polymorphism and coronary heart disease risk.**
(TIFF)

Figure S4 **Begg’s funnel plot of** ***RAGE*** **G82S polymorphism and coronary heart disease risk.**
(TIFF)

Table S1 **Minor allele distribution in cases and controls.**
(DOC)

Table S2 **Pairwise polymorphisms LD analysis.**
(DOC)

Table S3 **Meta analysis of haplotype combinations between −429T/C, −374T/A, and G82S polymorphisms of** ***RAGE*** **gene and CHD risk.**
(DOC)

Checklist S1 **PRISMA Checklist.**
(DOC)
