# Supplementary figures and images for: Meta-Analysis of RAGE Gene Polymorphism and Coronary Heart Disease Risk
Source: PLoS One. 2012 Dec 6;7(12):e50790. doi: 10.1371/journal.pone.0050790 (PMC3516500; doi:10.1371/journal.pone.0050790)

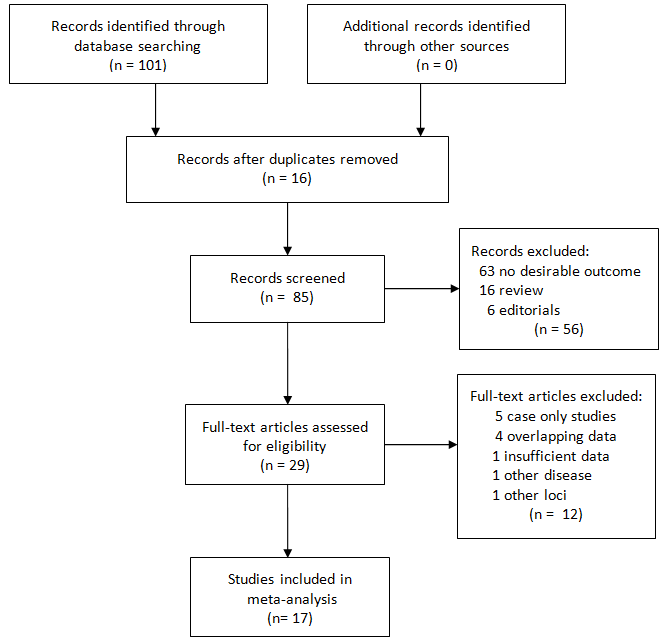

Supplement: Figure S1 — The flow chart of the included studies. (TIFF) [file pone.0050790.s001.tiff]

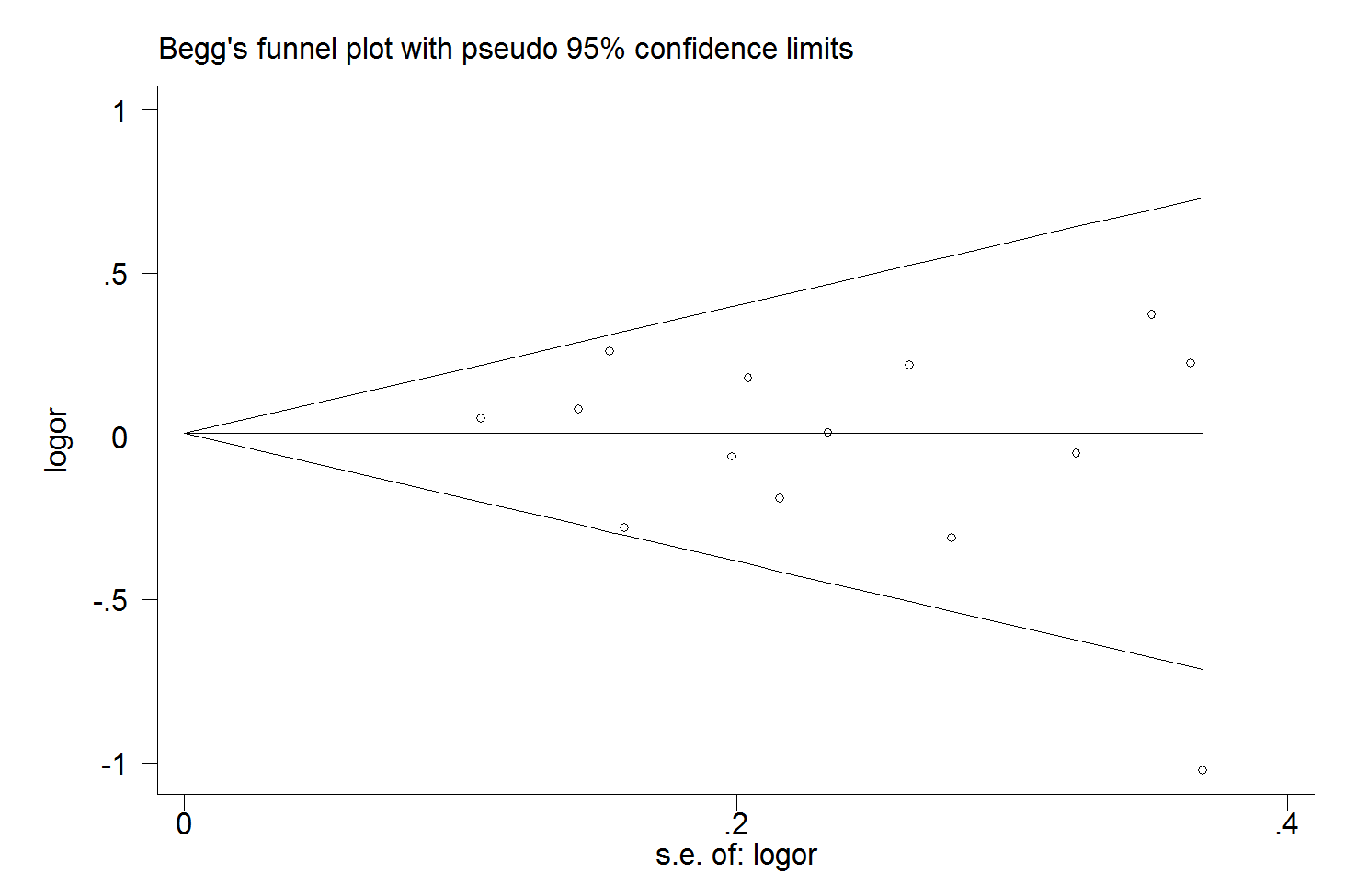

Supplement: Figure S2 — Begg’s funnel plot of RAGE −374T/A polymorphism and coronary heart disease risk. (TIFF) [file pone.0050790.s002.tiff]

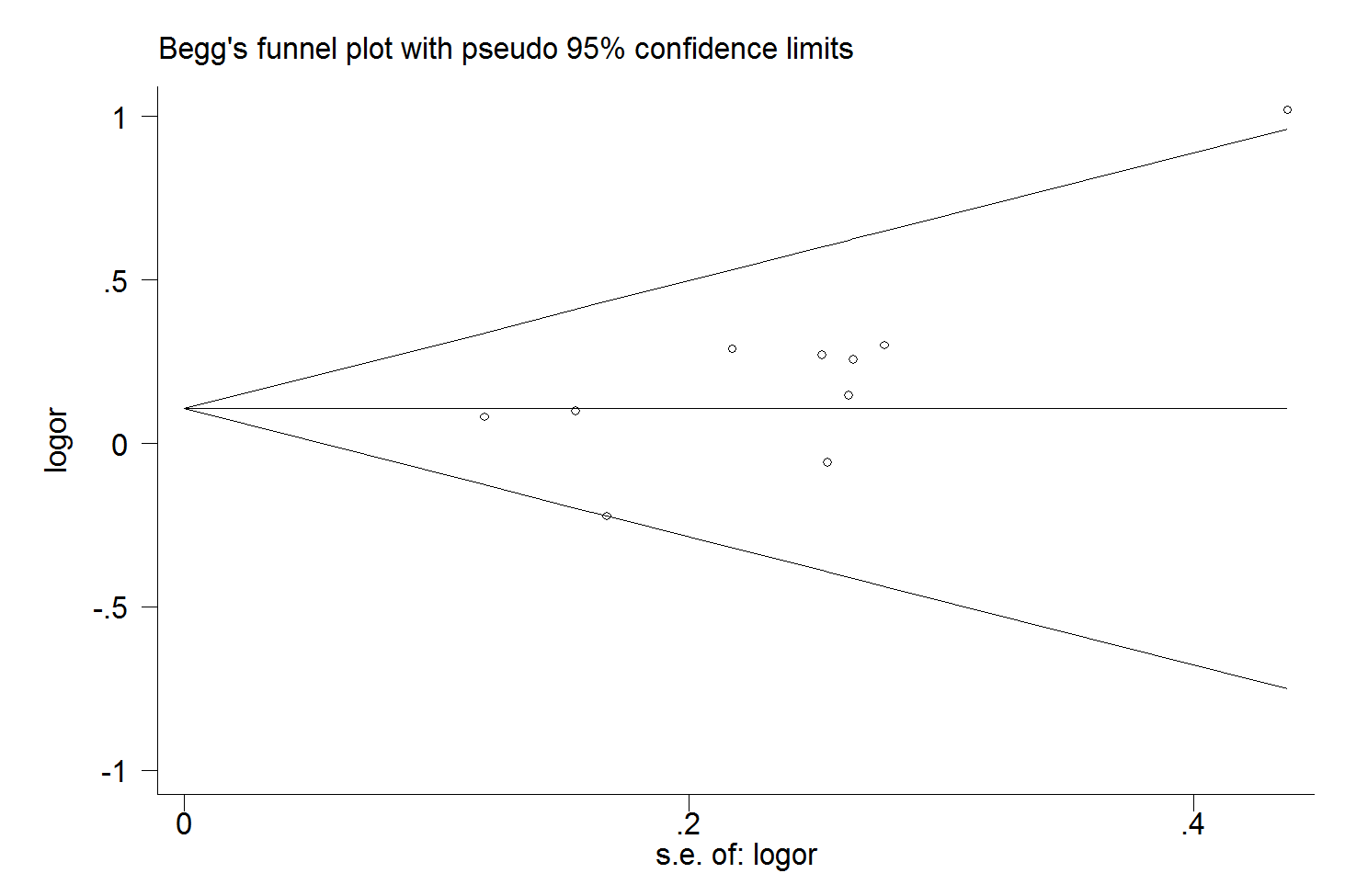

Supplement: Figure S3 — Begg’s funnel plot of RAGE −429T/C polymorphism and coronary heart disease risk. (TIFF) [file pone.0050790.s003.tiff]

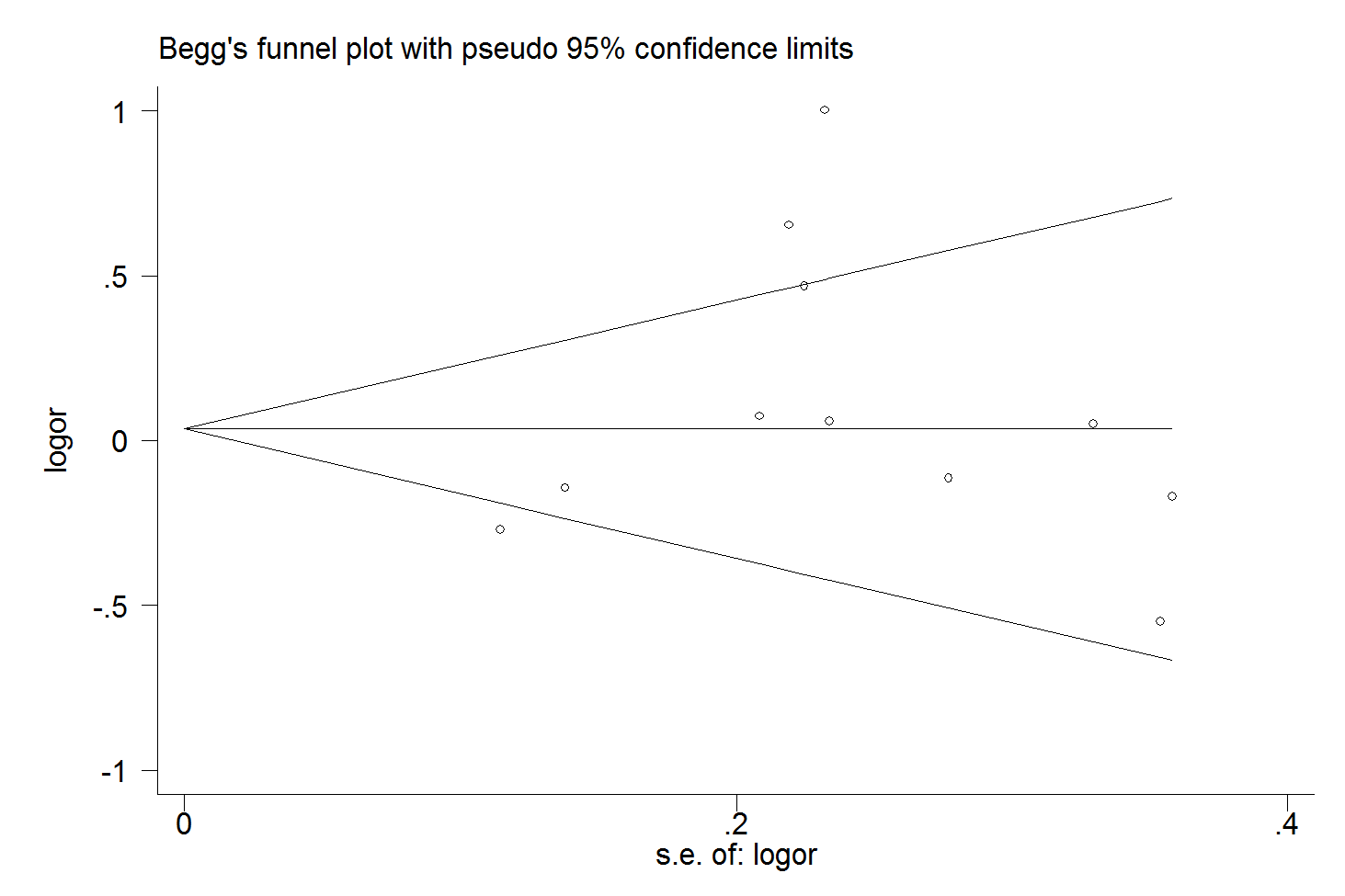

Supplement: Figure S4 — Begg’s funnel plot of RAGE G82S polymorphism and coronary heart disease risk. (TIFF) [file pone.0050790.s004.tif]
