# Supplementary material for: Meta-Analysis of RAGE Gene Polymorphism and Coronary Heart Disease Risk
Source: PLoS One. 2012 Dec 6;7(12):e50790. doi: 10.1371/journal.pone.0050790 (PMC3516500; doi:10.1371/journal.pone.0050790)
Supplement: Table S1 — Minor allele distribution in cases and controls. (DOC) [file pone.0050790.s005.doc]

| Study | Year | -429T/C MAF in cases/controls | -374T/A MAF in cases/controls | G82S MAF in cases/controls |
| --- | --- | --- | --- | --- |
| Pettersson-Fernholm | 2003 | NA | - | - |
| Falcone | 2004 | 0.35/0.45 | - | - |
| Kirbis | 2004 | 0.32/0.27 | 0.18/0.15 | - |
| Santos | 2005 | 0.26/0.31 | 0.14/0.10 | - |
| Hofmann | 2005 | - | - | 0.03/0.04 |
| Zee | 2006 | 0.28/0.25 | 0.15/0.19 | 0.04/0.04 |
| Yoon | 2007 | 0.20/0.19 | 0.13/0.12 | 0.13/0.16 |
| Lu | 2008 | 0.17/0.17 | 0.11/0.13 | 0.23/0.22 |
| Kucukhuseyin | 2009 | 0.49/0.37 | - | - |
| Peng | 2010 | 0.19/0.16 | 0.14/0.13 | 0.21/0.22 |
| Lu | 2010 | 0.14/0.12 | - | - |
| Xie | 2010 | 0.13/0.14 | - | - |
| Gao | 2010 | - | 0.12/0.10 | 0.24/0.15 |
| Kucukhuseyin | 2011 | - | - | 0.27/0.26 |
| Boiocchi | 2011 | 0.37/0.44 | - | - |
| Hou | 2011 | - | - | 0.46/0.40 |
| Aydogan | 2012 | 0.47/0.37 | - | 0.21/0.27 |

**Table S1.** Minor allele distribution in cases and controls.

MAF: minor allele frequency; NA: not available.
