# Supplementary material for: Meta-Analysis of RAGE Gene Polymorphism and Coronary Heart Disease Risk
Source: PLoS One. 2012 Dec 6;7(12):e50790. doi: 10.1371/journal.pone.0050790 (PMC3516500; doi:10.1371/journal.pone.0050790)
Supplement: Table S2 — Pairwise polymorphisms LD analysis. (DOC) [file pone.0050790.s006.doc]

**Table S2. Pairwise polymorphisms LD analysis.**

|  |  | r^2^ | | |
| --- | --- | --- | --- | --- |
|  |  | -429T/C | -374T/A | G82S |
| D’ | -429T/C | / | 0.048 | 0.006 |
|  | -374T/A | 1.00 | / | 0.007 |
|  | G82S | 1.00 | 1.00 | / |

Genotype data were downloaded from the International HapMap Project website (www.hapmap.org) for 30 CEPH trios. D’ indicates Lewontin’s normalized value; r, correlation coefficient.
