# Supplementary material for: Meta-Analysis of RAGE Gene Polymorphism and Coronary Heart Disease Risk
Source: PLoS One. 2012 Dec 6;7(12):e50790. doi: 10.1371/journal.pone.0050790 (PMC3516500; doi:10.1371/journal.pone.0050790)
Supplement: Table S3 — Meta analysis of haplotype combinations between −429T/C, −374T/A, and G82S polymorphisms of RAGE gene and CHD risk. (DOC) [file pone.0050790.s007.doc]

| Study | Ethnicity | Subjects | | TTG ^a^ | | TAG ^a^ | | CTG ^a^ | | TTS ^a^ | |
| --- | --- | --- | --- | --- | --- | --- | --- | --- | --- | --- | --- |
|  |  |  |  | Frequency | | Frequency | | Frequency | | Frequency | |
|  |  | Case | Control | Case | Control | Case | Control | Case | Control | Case | Control |
| Zee | American | 341 | 341 | 0.530 | 0.522 | 0.284 | 0.246 | 0.146 | 0.187 | 0.040 | 0.044 |
| Peng | Chinese | 370 | 370 | 0.491 | 0.505 | 0.162 | 0.161 | 0.135 | 0.123 | 0.207 | 0.207 |
| Yoon | Korean | 750 | 805 | 0.543 | 0.532 | 0.196 | 0.184 | 0.130 | 0.125 | 0.131 | 0.157 |
| Overall OR and 95 CI ^b^ | All | 1461 | 1516 | 1.01 (0.92-1.12) | | 1.10 (0.97-1.25) | | 0.96 (0.76-1.21) | | 0.88 (0.76-1.03) | |

**Table S2.** Meta analysis of haplotype combinations between -429T/C, -374T/A, and G82S polymorphisms of *RAGE* gene and CHD risk.

^a^ The haplotypes are those composed with -429T/C, -374T/A, and G82S polymorphisms. Only haplotypes with a frequency greater than 3% are listed.

^b^ Pooled from random effect model.

NA: not available

MAF: minor allele frequency; NA: not available.
